# 

**Published:** 1915-12-18

**Authors:** 


### CONTENTS.

PAGE

**Set Your Shoulder at the Wheel, to Advance the Business** ... ... ... ... ... ... 235

**Remember Absent Friends** ... ... ... ... 236

**Hospital and Institutional News** ... ... ... 237

A New Yorkshire Infirmary—An Institutional Will—Medical Inspection in War-Time—Trench Foot Again — Women Motor Ambulance Drivers—Important Massage Departments—The "Lady Soldier" on the English Genius—Permanent Buildings at £29 per Bed—Infant Care at Leeds—The Scruples of the Local Government Board—Unfit Recruits—A New Welsh War Hospital—The Notification of Measles—The Cost of Butter and Milk—The Billeting of Troops in Rural Areas—Municipal Grants to Voluntary Hospitals—The Lord Mayor of Leeds and its Hospitals—Glasgow Corporation's Strange Proposal—The New Supply Depôt at Bradford—The Coroner and Economy—An Antipodean Innovation—The Billeting System at Bournemouth—The Welsh Commissioners and a Panel Doctor—A Doctor Sues for his Salary as House Surgeon—A Suggested Amalgamation Scheme at Plymouth—Leeds' Latest Hospital Scheme—This Week's Drug Market.

**Lord Derby's Grand Effort** ... ... ... 243

The Medical Profession and Recruiting.

**Will Each Reader Please Note?** ... ... ... 244

**Some Older London Hospitals** ... ... ... ... 244

How They have been Re-created.

**The New King's College Hospital, London** (*illustrated*) ... ... ... ... ... ... 245

**Educational Value of Mr. Pite's Creation** ... 247

**War Disadvantages at King's College Hospital** 252

**Work and Economy in War-Time** ... ... 253

How to Secure Both by Co-operation.

**Hospital Needs and Hospital Appeals** ... ... 254

**Textile Supplies for Hospitals** ... ... ... 255

Chapter I.—With Subjects still to be Dealt With.

**Every Hospital's Own Gazette** (*illustrated*) ... 257

A Great Success at the Military Hospitals.

**Patriotism and Personal Service** ... ... ... 261

How the Giving Public were Recruited.

**A Year's Work at an Auxiliary Hospital** (*illustrated*) ... ... ... ... ... ... 262

The Record of the Arnold Hospital, Doncaster.

**The Hospital's Example in War-Time** ... ... 263

A More Excellent Way.

**Our Voluntary Hospitals and Institutions** ... 265

**"The Population Problem"** ... ... ... ... 265

**A Layman's Account of a Ward's Equipment** 266

What He found at University College Hospital.

**A War Hospital Supply Depot** ... ... ... 267

